# Medical, Environmental, and Social Determinants Associated With Periocular Cutaneous Malignancies in the United States Using the All of Us National Database

**DOI:** 10.7759/cureus.65831

**Published:** 2024-07-31

**Authors:** Niloofar Radgoudarzi, Liane Dallalzadeh, Bharanidharan R Saseendrakumar, Joy Guo, William Halfpenny, Don O Kikkawa, Sally Baxter

**Affiliations:** 1 Division of Ophthalmology Informatics and Data Science, Viterbi Family Department of Ophthalmology and Shiley Eye Institute, University of California San Diego, La Jolla, USA; 2 Division of Oculofacial Plastic and Orbital Surgery, Department of Ophthalmology, University of Texas Southwestern Medical Center, Dallas, USA; 3 Health Department of Biomedical Informatics, University of California San Diego, La Jolla, USA; 4 Division of Oculofacial Plastic and Reconstructive Surgery, Viterbi Family Department of Ophthalmology and Shiley Eye Institute, University of California San Diego, La Jolla, USA

**Keywords:** allofus national database, health inequities, social determinants of health, periocular cutaneous malignancy, big data

## Abstract

Objective: To identify common factors associated with periocular cutaneous malignancies using the National Institutes of Health (NIH) *All of Us* database.

Methodology: In this case-control study, we extracted electronic health records and sociodemographic data for 385 cases of periocular cutaneous malignancies from the *All of Us* nationwide database. Controls (*N* = 1540) were matched to the demographic characteristics of the 2020 United States Census. Bivariate analyses and multivariable logistic regression determined variables significantly associated with increased odds of periocular cutaneous malignancies. We analyzed medical, environmental, and social determinants to evaluate which factors were associated with increased odds of periocular cutaneous malignancies.

Results: Among the cases, the mean (standard deviation) age was 66.8 (11.2) years at the time of diagnosis. The majority were male (207, 54%) and white (361, 94%). Periocular cutaneous malignancy was significantly more likely among individuals with high sun exposure (odds ratio [OR] 14.79, 95% confidence interval [CI] 3.35-85.73, *P *= 0.001), those identifying as white race (OR 3.88, 95% CI 1.06-25.33, *P *= 0.079), and those with higher socioeconomic status, including higher annual income (OR 1.35, 95% CI 1.25-1.46, *P *< 0.001).

Conclusions: This study demonstrates similar risk factors for periocular cutaneous malignancies, echoing prior research that showed increased associations with lighter-pigmented skin and higher socioeconomic status. It also sheds light on the positive impact of physician surveillance and health utilization factors in the early detection and treatment of these malignancies, aspects less explored in prior analyses.

## Introduction

Cutaneous malignancies are the most common type of malignancy [1], among which about 10% occur in the periocular area [2]. The most common periocular skin tumor types are basal cell carcinoma (BCC), squamous cell carcinoma (SCC), and cutaneous melanoma (CM) [3]. Previous studies have demonstrated an increased incidence of all three tumor types among patients with lightly pigmented skin and higher socioeconomic status [4-6]. However, while patients of lower socioeconomic status and/or heavily pigmented minorities have a lower incidence of periocular cutaneous malignancies, they have a greater risk of poor clinical outcomes, including increased rates of advanced disease at presentation and higher stage-specific mortality rates [3,7-14].

The All of Us Research Program is an unprecedented effort by the National Institutes of Health (NIH) to collect and study data from 1 million adult participants across the United States. The program, launched in May 2018, prioritizes the enrollment of underrepresented minorities reflecting the increasing diversity of the US population [15,16]. The database provides electronic health record (EHR) and survey data for more than 736,000 adult participants to date [17], and enrollment is ongoing. This case-control study leverages the diversity and scale of the All of Us database to identify medical, environmental, and social factors associated with periocular cutaneous malignancies. Prior studies on these malignancies have been primarily single-center studies that were limited in sample size and/or diversity. Therefore, leveraging the large population, nationwide scope, and diversity of All of Us provides a unique opportunity to contribute to knowledge regarding this group of malignancies, which carry a substantial public health burden. This work was presented as a poster in the 53rd Annual Fall Scientific Symposium of the American Society of Ophthalmic Plastic and Reconstructive Surgery.

## Materials and methods

Data source and study population

The All of Us database contains EHR data, surveys, physical measurements, biospecimens, and wearable device data. EHR data about medical conditions, procedures, labs, and measurements are linked for all consented participants. Participants provide written informed consent and fill out a basic demographic survey upon enrollment covering factors such as education level, household income, and employment status [18]. Initial participant data collection was approved by the All of Us Institutional Review Board.

All the data have been transformed and de-identified across each participant record by the All of Us Program to protect participant privacy before sharing data with researchers on the All of Us Researcher Workbench. These transformations include data suppression of codes with a high risk of identification and generalization of categories such as age, sex at birth, gender identity, sexual orientation, and race. Secondary analyses of de-identified data included in All of Us, such as that presented here, are considered non-human subjects research and this was verified by the University of California Institutional Review Board. The study adhered to the Declaration of Helsinki 

There were 479,000 adult participants enrolled in All of Us at the time of analysis in March 2022. Cases were defined as adult (aged 18 years or older) participants with qualifying International Classification of Diseases (ICD) or Systematized Nomenclature of Medicine (SNOMED) codes corresponding to basal cell carcinoma, squamous cell carcinoma, and melanoma of the eyelids, medial canthus, cheek, forehead, or temple (see Supplementary Digital Content [SDC], Appendix A).

A total of 385 cases of periocular cutaneous malignancies were identified. Controls (*N *= 1,540) were sampled from the general adult population in the All of Us database with a 4:1 control-to-case ratio. Controls were sampled such that their demographics matched the 2020 US Census data about race, ethnicity, and gender using the R package MatchIt [19]. This was done to ensure that controls were representative of the general US population, given the known enrichment of minorities in the All of Us Program given its emphasis on enrolling individuals from populations traditionally underrepresented in biomedical research. A 4:1 control-to-case ratio was chosen to minimize the effects of class imbalance, and prior studies have shown that ratios higher than this do not confer additional statistical power [20].

Statistical analyses

Descriptive statistics of both cases and controls in the study cohort were generated (Table 1). We used mean/standard deviation for continuous variables and counts/percentages for categorical variables. We also examined the prevalence of periocular cutaneous malignancies by geographical region, as All of Us provided state-level data regarding the location of each study participant’s enrollment site.

**Table 1:** Demographic characteristics of adults with periocular cutaneous malignancies in the NIH All of Us research program and controls matched to the 2020 United States census. *Counts less than 20 are not disclosed, and additional data coarsening may be used to prevent secondary calculations of counts under 20, in accordance with All of Us data reporting policies.*

	Periocular cutaneous malignancies cases (*N* = 385)	Controls (*N* = 1,540)
Self-reported race, *N* (%)		
White	361 (94)	1175 (76)
Other	21 (5)	69 (4)
Asian	<20 (<5)*	90 (6)
Black or African American	<20 (<5)*	206 (13)
Self-reported ethnicity, *N *(%)		
Non-Hispanic or Latino	>360 (>95%)	1256 (82)
Hispanic or Latino	<20 (<5)*	284 (18)
Gender		
Male	207 (54)	758 (49)
Female	178 (46)	782 (51)
Mean age (SD) in years (at the time of diagnosis)	66.81 (11.16)	58.69 (17.45)
Mean age (SD) in years (at the time of the survey)	71.93 (11.26)	58.69 (17.45)

We performed bivariate and multivariable logistic regression using a wide range of predictors to identify medical, environmental, and social determinants significantly associated with increased odds of periocular cutaneous malignancies. Predictor variables included hypertension, hyperlipidemia, glaucoma, number of medical specialist visits, sun exposure (i.e., events of exposure to excess sunlight and to man-made ultraviolet light), geographical location, and sociodemographic variables (i.e., race/ethnicity, gender, employment status, insurance type, annual income). Correlation coefficients were generated to identify highly correlated variables to address any issues due to potential multi-collinearity in the multivariable modeling. Since none of the correlation coefficients exceeded 0.9, we proceeded with statistical modeling using the full complement of predictor variables. 

Bivariate analyses were performed to determine statistically significant variables. Bivariate odds ratios (OR) and 95% confidence intervals (CIs) were calculated for all predictors. A multivariable logistic regression model was then generated using bidirectional stepwise feature selection to identify variables significantly associated with increased odds of periocular cutaneous malignancy while adjusting for comparison of multiple variables simultaneously. Statistical significance was defined as *P *<= 0.05. All statistical analyses were performed in an R notebook within the All of Us Researcher Workbench environment, which is accessible upon request to all registered All of Us users [21].

## Results

A total of 385 cases of periocular cutaneous malignancy were identified (Table 1). The mean (standard deviation) age was 66.8 (11.2) years at the time of diagnosis, and 71.9 (11.3) years at the time of survey. The majority of cases were male (207, 53.8%) and white (361, 93.8%). BCC, SCC, and melanoma accounted for 70.02%, 25.36%, and 4.20% of the cases, respectively, and the remaining 0.42% had sebaceous adenocarcinoma.

Bivariate analyses to identify predictors associated with increased odds of periocular cutaneous malignancy demonstrated a significant association with white race (crude OR 13.83, 95% CI 4.35-84.19, *P *< 0.001), higher education level (OR 1.82, 95% CI 1.57-2.13, *P *< 0.001), higher annual income (OR 1.37, 95% CI 1.31-1.43, *P *< 0.001), and current homeownership (OR 2.72, 95% CI 1.74-4.48, *P *< 0.001). In addition, medical conditions, including hyperlipidemia (OR 4.93, 95% CI 3.89-6.27, *P *< 0.001), hypertension (OR 3.45, 95% CI 2.74-4.35, *P *< 0.001), macular degeneration (OR 3.65, 95% CI 2.12-6.24, *P *< 0.001), and glaucoma (OR 1.63, 95% CI 1.09-2.40, *P *= 0.015), and environmental conditions such as sun exposure (OR 22.22, 95% CI 7.35-95.87, *P *< 0.001) were associated with increased risks of periocular cutaneous malignancy. Those with poor social satisfaction (OR 0.18, 95% CI 0.06-0.41, *P *< 0.001; see SDC, Appendix B) and concerned with stable housing (OR 0.22, 95% CI 0.13-0.35, *P *< 0.001) were associated with lower risk (Table 2).

**Table 2:** Bivariate analyses of odds ratios for variables significantly associated with increased odds of periocular cutaneous malignancies.

Variable	Odds ratio (OR)	95% confidence interval (CI)	*P*-value
Sun exposure	22.22	7.35-95.87	<0.001
White race	13.83	4.35-84.19	<0.001
Education level	1.82	1.57-2.13	<0.001
Annual income	1.37	1.31-1.43	<0.001
Current homeowner	2.72	1.74-4.48	<0.001
Hyperlipidemia	4.93	3.89-6.27	<0.001
Hypertension	3.45	2.74-4.35	<0.001
Macular degeneration	3.65	2.12-6.24	<0.001
Glaucoma	1.63	1.09-2.40	0.015
Poor social satisfaction	0.18	0.06-0.41	<0.001
Concerned with stable housing	0.22	0.13-0.35	<0.001

**Figure 1:**
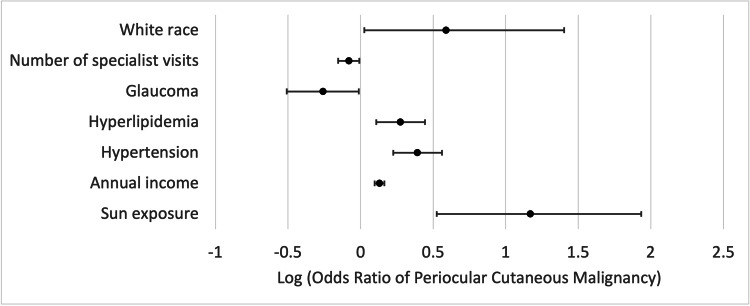
Odds ratios (OR) associated with periocular cutaneous malignancies derived from the multivariable models. Log of OR is used for scaling purposes and better visualization. Note that log(OR) > 0 indicates greater associated risk.

**Figure 2:**
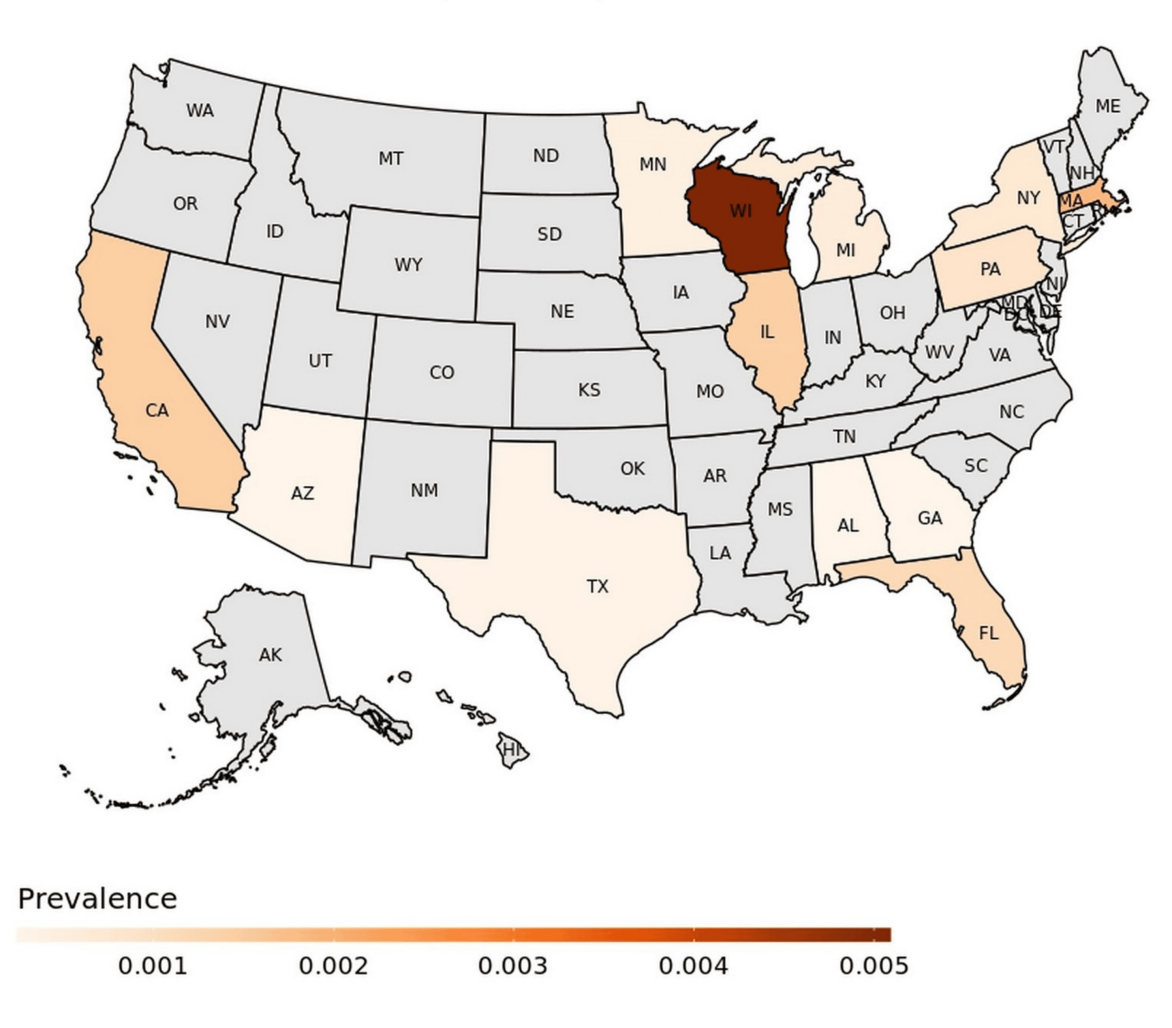
Periocular cutaneous malignancy prevalence in each state among All of Us participants.

In multivariable analyses, medical conditions, including hypertension (adjusted OR 2.46, 95% CI 1.68-3.63, *P *< 0.001) and hyperlipidemia (OR 1.88, 95% CI 1.28-2.78, *P *= 0.001), and environmental conditions such as sun exposure (OR 14.79, 95% CI 3.35-85.73, *P *= 0.001) remained significantly associated with increased risk of periocular cutaneous malignancy. Glaucoma (OR 0.55, 95% CI 0.31-0.97, *P *= 0.039) and higher number of specialist visits (OR 0.83, 95% CI 0.70-0.98, *P *= 0.028) were linked with reduced risk (Table 3 and Figure 1). Wisconsin, Massachusetts, California, and Illinois, with 0.50%, 0.19%, 0.14%, and 0.14% prevalence, respectively, demonstrated the highest prevalence of periocular cutaneous malignancies among all states (Figure 2).

**Table 3:** Adjusted odds ratios for variables significantly associated with increased odds of periocular cutaneous malignancies using multivariable logistic regression.

Variable	Odds ratio (OR)	95% confidence interval (CI)	*P*-value
Sun exposure	14.79	3.35-85.73	0.001
Annual income	1.35	1.25-1.46	<0.001
Hypertension	2.46	1.68-3.63	<0.001
Hyperlipidemia	1.88	1.28-2.78	0.001
Glaucoma	0.55	0.31-0.97	0.039
Number of specialist visits	0.83	0.70-0.98	0.028
White race	3.88	1.06-25.33	0.079

## Discussion

In this study, we aimed to leverage a diverse nationwide database to better understand risk factors associated with periocular cutaneous malignancies, including basal cell carcinoma, squamous carcinoma, melanoma, and sebaceous adenocarcinoma. While a previous review study did show an increased incidence of periocular cutaneous malignancies in patients with lightly pigmented skin and higher socioeconomic status [3], it was conducted in a population group with limited diversity. Our study is unique in that it leverages a novel nationwide database with demographic data from a diverse population, including underrepresented minorities.

Similar to prior studies, our bivariate analyses showed a significant association between white race and periocular cutaneous malignancy. Part of this can be explained by biological differences in melanin concentration and associated ultraviolet (UV) protection in light versus heavier-pigmented skin types. In addition, our study showed that race and ethnicity are closely intertwined with socioeconomic factors such as educational level and annual income. Our bivariate analyses demonstrated that higher education level, annual income, and homeownership were associated with increased risk, while those with poor social satisfaction and those concerned with stable housing had lower periocular cutaneous malignancy risk. Higher-income remained significantly associated with greater risk in the multivariable model when accounting for other factors such as medical comorbidities (adjusted OR 1.35, 95% CI 1.25-1.46, *P *< 0.001), whereas white race was borderline significant when other factors were accounted for (adjusted OR 3.88, 95% CI 1.06-25.33, *P *= 0.08). This is in contrast with other conditions where, in general, lower socioeconomic status has typically been associated with greater disease risk, such as glaucoma and diabetic retinopathy [22,23]. Therefore, this represents a situation where disease risk is present even among those with relatively better resources, despite presumably better access to healthcare services.

A possible explanation for this may be related to behavioral differences between groups, with particular emphasis on recreational UV exposure [24]. For instance, those in high-socioeconomic groups in the United States likely have increased opportunity for more intense recreational sunlight exposure, potentially due to increased amount of leisure time, and/or increased resources for engaging in high-cost outdoor recreational activities. This was also demonstrated in our multivariable model, where sun exposure was the risk factor with the highest OR in association with malignancy (adjusted OR 14.79, 95% CI 3.35-85.73, *P *= 0.001). However, the incidence of periocular skin cancers can be reduced with the long-term use of sunscreens, sunglasses, and hats with brims [25]. Ophthalmologists should counsel patients who endorse substantial sun exposure about the risk of periocular cutaneous malignancy and advise them to take precautions accordingly. 

Further, sun exposure in patients who live and work in states with a large agricultural industry may have contributed to geographic differences in prevalence. Our data supports this hypothesis, as 3 out of 4 top states with the highest prevalence (California, Illinois, and Wisconsin) are also among the top 10 in agricultural production [26]. However, diagnostic bias may have also contributed to this, since Illinois and Massachusetts are among the states with the highest employment rates for ophthalmologists [27].

The multivariable analysis also suggests that a higher number of specialist visits is associated with a lower risk of periocular cutaneous malignancy. This is likely due to increased surveillance of the periocular area in these patients and with earlier detection and intervention leading to the reduced incidence in patients with concurrent glaucoma who visit an ophthalmologist more frequently. This is consistent with prior findings that skin cancer can be partially preventable by physician counseling, especially for high-risk patients with a current or previous history of nonmelanoma melanoma skin cancer, or actinic keratosis [28]. This data demonstrates the important role of ophthalmologists in the detection and treatment of skin cancer. Even ophthalmologists who subspecialize in intraocular pathology should remember to examine the eyelid and the ocular adnexal regions and have a low threshold to refer patients with suspicious lesions to oculoplastics specialists and/or dermatologists for further evaluation.

This study has some limitations. First, diagnoses of periocular cutaneous malignancies were based on diagnostic and billing codes captured in the All of Us database, rather than clinical notes, photographs, or pathology reports. Therefore, we could not verify the diagnoses clinically. Similarly, without clinical notes, we could not ascertain the treatment course. Finally, exact details about actual sun exposure and recreational activities were somewhat limited, as we were constrained by the existing survey instruments in All of Us. As with all observational studies, statistical associations do not imply causation. However, despite these limitations, this data source does offer a large sample size, multi-site enrollment across the United States, and variables regarding healthcare access and utilization and socioeconomic characteristics, which are typically not readily available in routine claims data or other EHR databases.

## Conclusions

Our analyses of All of Us nationwide data demonstrate a significant correlation between cutaneous periocular tumors and certain medical, environmental, and social factors. Increased risk of these malignancies was seen in individuals of higher socioeconomic status with greater sun exposure. Although higher socioeconomic status typically comes with better access to healthcare and general well-being, those individuals are also more likely exposed to recreational outdoor activities with extended sun exposure. Our findings validate those of prior studies now utilizing a national diverse database and serve to inform ophthalmologists of the patients who require close monitoring regarding the development of periocular malignancies.

## Appendices

Appendix A 

**Table 4:** Supplementary digital content: criteria codes.

Criterion	Concept ID	SNOMED code
Basal cell carcinoma of the cheek	4292381	402507001
Basal cell carcinoma of the eyelid	4335883	231832009
Basal cell carcinoma of the forehead	4298033	403916003
Basal cell carcinoma of the lower eyelid	4291143	402494003
Basal cell carcinoma of the medial canthus	4292378	402495002
Basal cell carcinoma of the temple	4300565	403917007
Basal cell carcinoma of the upper eyelid	4297185	403919005
Exposure to excess sunlight	4053945	242535001
Malignant melanoma of the eye	4170619	274087000
Malignant melanoma of the eyelid	4338760	231834005
Malignant melanoma of the skin of the cheek	4244166	93217003
Malignant melanoma of the skin of the eyelid	434590	93224002
Malignant melanoma of skin of lower eyelid	4309539	423447006
Melanoma in situ of eyelid, including canthus	4033836	109274007
Primary basal cell carcinoma of left eyelid	36712713	1079111000119108
Primary basal cell carcinoma of right eyelid	36712715	1079171000119100
Sebaceous adenocarcinoma of eyelid	4335884	231833004
Squamous cell carcinoma of eyelid	4338759	231831002
Squamous cell carcinoma of forehead	4300556	403893006
Squamous cell carcinoma of skin of cheek	4111221	285309005
Squamous cell carcinoma of temple	4314299	425148008

Appendix B

**Table 5:** Supplementary digital content: relevant survey questions.

Category	Survey question	Answer choices
Social satisfaction	In general, how would you rate your satisfaction with your social activities and relationships?	Excellent, very good, good, fair, poor, skip
Stable housing	In the past 6 months, have you been worried or concerned about NOT having a place to live?	Yes, no, skip

## References

[1] Simões MC, Sousa JJ, Pais AA (2015). Skin cancer and new treatment perspectives: a review. Cancer Lett.

[2] Cook BE, Bartley GB (1999). Epidemiologic characteristics and clinical course of patients with malignant eyelid tumors in an incidence cohort in Olmsted county, Minnesota. Ophthalmology.

[3] Broadbent T, Bingham B, Mawn LA (2016). Socioeconomic and ethnic disparities in periocular cutaneous malignancies. Semin Ophthalmol.

[4] Gloster HM Jr, Neal K (2006). Skin cancer in skin of color. J Am Acad Dermatol.

[5] Harris RB, Griffith K, Moon TE (2001). Trends in the incidence of nonmelanoma skin cancers in southeastern Arizona, 1985-1996. J Am Acad Dermatol.

[6] Cormier JN, Xing Y, Ding M (2006). Ethnic differences among patients with cutaneous melanoma. Arch Intern Med.

[7] Galindo-Ferreiro A, Sanchez-Tocino H, Diez-Montero C (2020). Characteristics and recurrence of primary eyelid basal cell carcinoma in central Spain. J Curr Ophthalmol.

[8] Lim LT, Agarwal PK, Young D, Ah-Kee EY, Diaper CJ (2015). The effect of socio-economic status on severity of periocular basal cell carcinoma at presentation. Ophthalmic Plast Reconstr Surg.

[9] Robinson JK, Altman JS, Rademaker AW (1995). Socioeconomic status and attitudes of 51 patients with giant basal and squamous cell carcinoma and paired controls. Arch Dermatol.

[10] Kakagia D, Trypsiannis G, Karanikas M, Mitrakas A, Lyratzopoulos N, Polychronidis A (2013). Patient-related delay in presentation for cutaneous squamous cell carcinoma. A cross-sectional clinical study. Onkologie.

[11] Singh GK (2003). Area Socioeconomic Variations in US Cancer Incidence, Mortality, Stage, Treatment, and Survival, 1975-1999. National Cancer Institute.

[12] Reyes-Ortiz CA, Goodwin JS, Freeman JL (2005). The effect of socioeconomic factors on incidence, stage at diagnosis and survival of cutaneous melanoma. Med Sci Monit.

[13] Harvey VM, Patel H, Sandhu S, Wallington SF, Hinds G (2014). Social determinants of racial and ethnic disparities in cutaneous melanoma outcomes. Cancer Control.

[14] Geller AC, Miller DR, Lew RA, Clapp RW, Wenneker MB, Koh HK (1996). Cutaneous melanoma mortality among the socioeconomically disadvantaged in Massachusetts. Am J Public Health.

[15] Mapes BM, Foster CS, Kusnoor SV (2020). Diversity and inclusion for the All of Us research program: a scoping review. PLoS One.

[16] Denny JC, Rutter JL, Goldstein DB, Philippakis A, Smoller JW, Jenkins G, Dishman E (2019). The "All of Us" research program. N Engl J Med.

[17] (2022). All of Us Data Snapshots. https://www.researchallofus.org/data-tools/data-snapshots/.

[18] (2022). All of Us Participants Surveys: The Basics. https://www.researchallofus.org/data-tools/survey-explorer/the-basics-survey/..

[19] Ho D, Imai K, King G, Stuart EA (2011). MatchIT: Nonparametric preprocessing for parametric causal inference. J Stat Software.

[20] Gail M, Williams R, Byar DP, Brown C (1976). How many controls?. J Chron Diseases.

[21] (2020). Health Disparities in Periocular Malignancies Workspace. https://workbench.researchallofus.org/workspaces/aou-rw-83ce016f/ophthalmologyanddisparities/data..

[22] Chan AX, McDermott Iv JJ, Lee TC, Ye GY, Shahrvini B, Saseendrakumar BR, Baxter SL (2022). Associations between healthcare utilization and access and diabetic retinopathy complications using All of Us nationwide survey data. PLoS One.

[23] Lee TC, Saseendrakumar BR, Nayak M (2022). Social determinants of health data availability for patients with eye conditions. Ophthalmol Sci.

[24] Reyes-Ortiz CA, Goodwin JS, Freeman JL, Kuo YF (2006). Socioeconomic status and survival in older patients with melanoma. J Am Geriatr Soc.

[25] Margo CE, Waltz K (1993). Basal cell carcinoma of the eyelid and periocular skin. Surv Ophthalmol.

[26] (2022). Ranking of States That Produce the Most Food. https://beef2live.com/story-states-produce-food-value-0-107252.

[27] (2022). U.S. Bureau of Labor Occupational Employment and Wage Statistics. https://www.bls.gov/oes/current/oes291241.htm.

[28] Feldman SR, Fleischer AB Jr (2000). Skin examinations and skin cancer prevention counseling by US physicians: a long way to go. J Am Acad Dermatol.

